# P-1704. Impact of an Empiric Antibiotic Pocket Guide on Rates of Guideline-concordant Empiric Regimens for Inpatient Community-acquired Pneumonia at a Community Hospital

**DOI:** 10.1093/ofid/ofae631.1870

**Published:** 2025-01-29

**Authors:** Elizabeth Bernauer, Martin Brenneman, Taylor H Johnson

**Affiliations:** Baptist Health Hardin, Louisville, Kentucky; Baptist Health Hardin, Louisville, Kentucky; Baptist Health Hardin, Louisville, Kentucky

## Abstract

**Background:**

Community-acquired pneumonia (CAP) is a common cause of hospital admissions and studies have shown concordance with guideline recommendations improves outcomes. Antibiotic pocket guides (PG) have been shown to increase use of preferred agents in urinary tract and intra-abdominal infections, but their impact on inpatient CAP treatment has not yet been evaluated. This study sought to evaluate the impact of a newly introduced empiric antibiotic PG (introduced May 2023) on rates of guideline-concordant empiric CAP regimens within 24 hours of admission.

**Figure:**
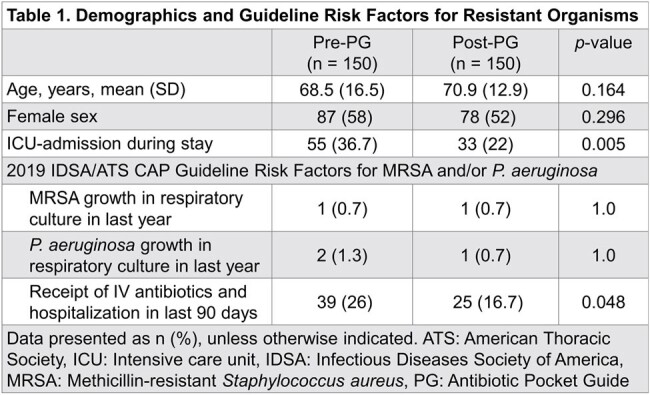

**Methods:**

This retrospective, pre-post, quasi-experimental study conducted at a 300-bed community hospital included adult inpatients initiated on antibiotics with order indication of pneumonia (+/- sepsis) within 24 hours. Patients were excluded if other indications were selected, if they had severe β-lactam allergies precluding use of all recommended agents, or if initiated on azithromycin or doxycycline alone. Included patients were randomly stratified 1:1 from pre-PG (Jul–Sep 2022) and post-PG (Jul–Sep 2023). The primary outcome was the rate of guideline-concordant empiric antibiotic selection for CAP between the two time periods. Secondary outcomes included total duration of therapy and *C. difficile* rate within 60 days of admission. Length of stay and all-cause mortality within 30 days of discharge were exploratory safety outcomes.

**Figure:**
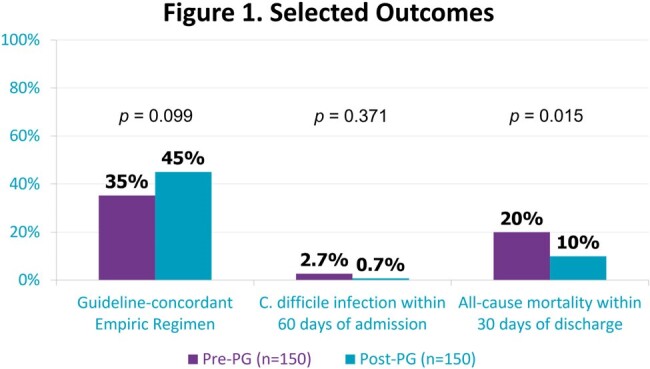

**Results:**

300 patients were included,150 per group. Baseline demographics and risk factors were similar between groups except for higher rates of ICU-admission and recent hospitalization with IV antibiotics in the pre-PG group (Table 1). Patients received a guideline-concordant regimen more frequently post-PG than pre-PG, but this did not reach statistical significance (44.7% vs 35.3%, p = 0.099). No difference in appropriate duration was observed. 30-day all-cause mortality was significantly higher in the pre-PG group (p = 0.015). Other details on management and outcomes can be seen in Table 2 and Figures 1-2.

**Figure:**
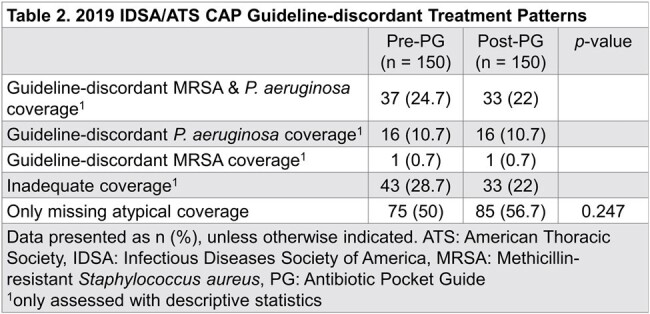

**Conclusion:**

Introduction of an antibiotic pocket-guide did not result in a significant change in guideline-concordant empiric CAP treatment in this study. Though no evidence of harm was observed, other interventions may be needed to improve guideline-concordant inpatient CAP treatment.

**Figure 2.**
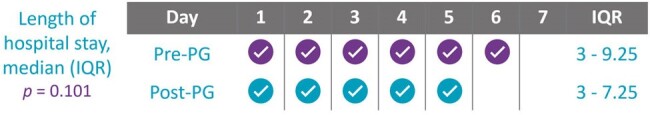
Hospital Length of Stay

**Disclosures:**

**Martin Brenneman, PharmD, BCIDP**, Cardinal Health, Inc.: Stocks/Bonds (Public Company)|Dexcom, Inc: Stocks/Bonds (Public Company)|Insulet Corp.: Stocks/Bonds (Public Company)|Johnson & Johnson: Stocks/Bonds (Public Company)|Medtronic Plc: Stocks/Bonds (Public Company)

